# Cuproptosis-related LncRNAs are correlated with immunity and predict prognosis in HNSC independent of TMB

**DOI:** 10.3389/fgene.2023.1028044

**Published:** 2023-02-01

**Authors:** Mingyu Li, Yeltai Nurzat, He Huang, Peiru Min, Xiaowen Zhang

**Affiliations:** ^1^ Department of Plastic and Reconstructive Surgery, Shanghai Ninth People’s Hospital Affiliated to Shanghai Jiao Tong University School of Medicine, Shanghai, China; ^2^ State Key Laboratory of Respiratory Disease, Department of Otolaryngology, Head and Neck Surgery, Laboratory of ENT-HNS Disease, First Affiliated Hospital of Guangzhou Medical University, Guangzhou, China; ^3^ Department of Oral and Maxillofacial—Head and Neck Oncology, Shanghai Ninth People’s Hospital, Shanghai Jiao Tong University School of Medicine, College of Stomatology, Shanghai Jiao Tong University, National Center for Stomatology, National Clinical Research Center for Oral Diseases, Shanghai Key Laboratory of Stomatology, Shanghai Research Institute of Stomatology, Shanghai, China; ^4^ Department of Allergy and Clinical Immunology, The First Affiliated Hospital, Guangzhou Medical University, Guangzhou, China; ^5^ Department of Cancer, The First Affiliated Hospital, Guangzhou Medical University, Guangzhou, China

**Keywords:** head and neck squamous cell carcinoma, cuproptosis, lncRNA, prognostic model, tumor mutation burden

## Abstract

**Aims:** Cuproptosis is a novel cell death pathway, and the regulatory mechanism in head and neck squamous cell carcinoma (HNSC) remains to be explored. We determined whether cuproptosis-related lncRNAs (CRLs) could predict prognosis in HNSC.

**Methods and Results:** First, we identified 10 prognostic CRLs by Pearson correlation and univariate Cox regression analyses. Next, we constructed the CRLs prognostic model based on 5 CRLs screened by the least absolute shrinkage and selection operator (LASSO) Cox analysis. Following this, we calculated the risk score for HNSC patients and divided patients into high- and low-risk groups. In our prognostic model, HNSC patients with higher risk scores had poorer outcomes. Based on several prognostic features, a predictive nomogram was established. Furthermore, we investigated principal component analysis to distinguish two groups, and functional enrichment analysis of 176 differentially expressed genes (DEGs) between risk groups was performed. Finally, we analyzed relationships between tumor mutation burden (TMB) and risk scores.

**Conclusion:** Cuproptosis-related lncRNAs can be applied to predict HNSC prognosis independent of TMB, which is closely correlated with tumor immunity.

## Introduction

Head and neck squamous cell carcinoma (HNSC) is a common, multiple and often fatal malignant tumor, accounting for 5% of all malignancies per year. HNSC patients are mostly diagnosed in late stage and often have poor prognosis (Aggarwal et al., 2019). The availability of checkpoint inhibitors for metastatic HNSC has changed outcomes for this disease but durable benefit and survival gains occur only in a small subset of patients (Ferris et al., 2016; Bauml et al., 2017). Recently, associations between molecular biological biomarkers and tumor prognosis during the development of HNSC have elicited great interest (Ludwig et al., 2017). However, our understanding of the etiology and pathogenesis of HNSC could be improved, and more effective prognostic biomarkers are required.

In March 2022, Peter Tsvetkov and Todd R. Golub’s team found that copper-mediated cell death differed from known mechanisms, which was named cuproptosis (Tsvetkov et al., 2022). In the progression of tumor, cell death plays a key decisive role. For example, dysregulation of autophagy will lead to oncogenesis because of the aberrant activation of oncogenes such as PIK3CA and inactivation of tumor suppressor genes like PTEN (Kimmelman and White, 2017). Upon stressors like nutrient and oxygen deprivation, autophagy can promote tumor survival and growth *via* regulating tumor metabolism (Degenhardt et al., 2006). Although the relationships between other types of cell death and tumorigenesis were previously proved (Pavlyukov et al., 2018), oncological studies in cuproptosis were insufficient.

LncRNA plays an important role in tumor proliferation as well as migration, and is involved in tumor cell death (Yang et al., 2014; Peng et al., 2017). It was reported that lncRNA could enhance the invation and metastasis of HNSC. Moreover, lncRNA would potentially become prognosticator or therapeutic target in HNSC (Luo et al., 2018). However, the association between lncRNA and cuprotosis in HNSC needs further study. If we can explain and discover the role of cuprotosis in HNSC, we are expected to provide new drug targets for clinical treatment of HNSC, or clarification of the pathogenesis.

## Materials and methods

### Datasets

The RNA sequencing data of HNSC and clinical characteristics were obtained from the TCGA database (https://portal.gdc.cancer.gov), including 111 HNSC patients and 12 normal pancreatic tissues (samples without expression matrix or clinical information were excluded). The expression of lncRNAs was extracted according to the human gene annotations in GENCODE (https://www.gencodegenes.org/). After log2 transformation, function avereps in limma package was performed to merge the overlapped data. For clinical data, we eliminated samples with missing values. Cuproptosis regulators were obtained from the previous study (FDX1, LIAS, LIPT1, DLD, DLAT, GLS,PDHA1, PDHB, MTF1, and CDKN2A) (Tsvetkov et al., 2022). TMB data was downloaded from TCGA using TCGAbiolinks package, and the tumor immune dysfunction and exclusion (TIDE) scoring file was retrieved from the TIDE website (http://tide.dfci.harvard.edu) (Jiang et al., 2018). The external validation datasets were obtained from Kaplan-Meier Plotter (http://kmplot.com/analysis/) and CESC data in TCGA (Nagy et al., 2021).

### Bioinformatic analysis

Our bioinformatic analysis was based on R (version 4.1.3) software. We identified CRLs by limma package in R (|Pearson R| > 0.4, *p* < 0.05). Then we obtained prognostic CRLs through the univariate Cox regression analysis (*p* < 0.05) with survival package. We performed Lasso cox regression analysis with glmnet package to construct the prognostic model. The risk score was calculated according to the formula: Risk score = Σ Coef ∗ EXP. In this formula, Coef is the coefficient and EXP is the expression level of each prognostic CRLs. HNSC patients were divided into high-risk group and low-risk group based on the value of risk scores. The cutoff value was depended by surv_cutpoint function in survminer package. Kaplan-Meier survival curves were drawn *via* survminer package as well. Corresponding ROC curves were drawn by timeROC package. DEGs between risk categories were filtered using limma package (|log2FoldChange| > 1, *p* < 0.05). The DEGs were performed GO and KEGG enrichment analyses *via* clusterProfiler package. A nomogram was constructed using the rms package.

### Immune infiltration analysis

After gene expression profile extraction and mapping on the GeneSymbol of each tumor, GSVA, GSEAbase and immunedeconv packages in R were used to give the immune function scores for each sample on the basis of gene expression. Seven different algorithms were performed including XCELL, QUANTISEQ, TIMER, EPIC, ESTIMATE, MCPCOUNTER, and ssGSEA. The online TIMER (Tumor Immune Estimation Resource) database (http://timer.cistrome.org/) was also exploited in our research (Li et al., 2017).

After calculation of infiltration value for immune or stromal cells, limma, reshape2 and pheatmap packages were used to obtain and visualize the immune infiltration signature of different risk groups.

### Statistical analysis

CRLs were identified by Pearson correlation test. To compare overall survival between subgroups, Kaplan-Meier (KM) analysis was used. The difference in risk scores between subgroups was compared using the student’s t-test, and categorical variables of groups were analyzed by chi-square test. The correlation among subtypes was calculated using the Pearson correlation test. We explored the independent prognostic value of the risk scores and other clinical features using univariate and multivariate Cox regression analyses. Statistical analysis was conducted by the “R” software. In our study, a *p*-value of less than 0.05 was considered statistically significant.

### Quantitative RT-PCR (qRT-PCR) analysis

Different HNSC cell lines (HN4, HN6, HN30, CAL27, SCC9, and SCC25) as experiment groups were obtained from Department of Head and Neck Surgery, Shanghai Ninth People’s Hospital for qRT-PCR. Human oral keratinocytes (HOK) were set as the normal control group. Total RNA was extracted using RNA-Quick Purification Kit (RN001, YiShan Biotech, China) according to the manufacturer’s protocol. Then, reverse transcription of RNA into cDNA was conducted using the Fast All-in-One RT Kit (with gDNA Remover) (RT001, YiShan Biotech, China). We performed qRT-PCR in LightCycler 96 (Roche, United States) using Hieff UNICON qPCR SYBR Green Master Mix (11198ES03, Yeasen, China). The lncRNA expression levels of LINC02178, LINC01473, MIR3945HG, LRRK2-DT and AL137804.1 were detected. PCR primers were designed based on sequences from the corresponding genes (Table 1). All data were normalized using ActinB as the internal control by the Δ-CT method.

**TABLE 1:** The primer sequences for qRT-PCR.

Primers	Sequences
LINC02178 Forward primer	5' CAG CAC GAG AGT TGT AGG CA 3'
LINC02178 Reverse primer	5' TTT AGC AAC ATC ACA GCG GC 3'
LINC01473 Forward primer	5' AGC AGG AAG AAG TAC AAG CAA AG 3'
LINC01473 Reverse primer	5' AGG GGA CAC ATG CCA AGG AT 3'
MIR3945HG Forward primer	5' GAA AGA AAC GCC CAC GTT GAG 3'
MIR3945HG Reverse primer	5' GAC TTG CGG GAG GAG AAT GT 3'
LRRK2-DT Forward primer	5' GCC CGC CTG TTT ATG AGG AA 3'
LRRK2-DT Reverse primer	5' CTC GTT TTT GGG GCC TGA GT 3'
AL137804.1 Forward primer	5' GGC TTG TTT GGC CTT CCA AT 3'
AL137804.1 Reverse primer	5' GTG CCC CAG CAT AGG GAT AG 3'

## Results

### Identification of prognostic CRLs and construction of CRLs prognostic model

**FIGURE 1:**
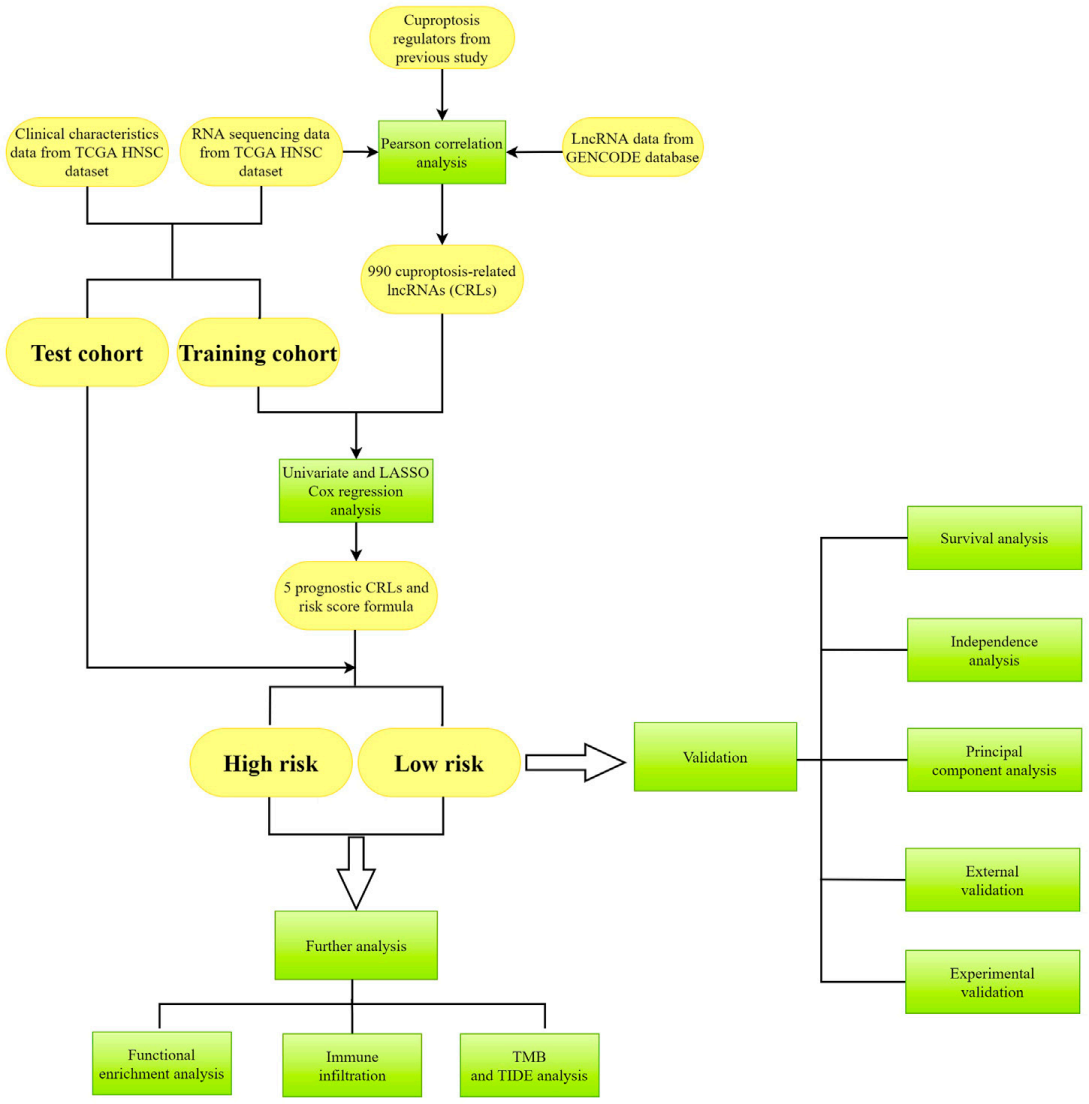
The flowchart of analysis in this study. Yellow color represents the data, and green color represents the methods.

**FIGURE 2:**
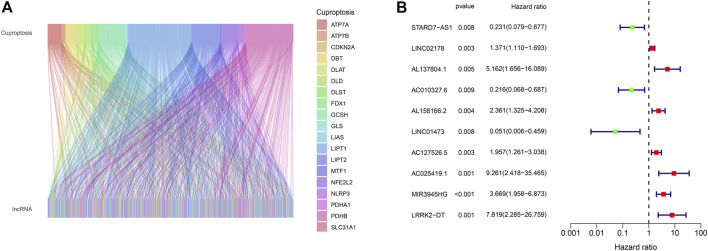
**(A)** Alluvial plot of pearson correlation analysis between 990 CRLs and 19 cuproptosis regulators. **(B)** The hazard ratio and expression of 10 prognostic CRLs.

The whole process of our analysis was shown in the flowchart (Figure 1). Firstly, we downloaded the head and neck squamous cell carcinoma cancer datasets from TCGA database, which included 111 tumor samples and 12 normal samples (samples without expression matrix or clinical information were excluded). According to the GENCODE database, we identified 16,876 lncRNAs from TCGA HNSC dataset. According to the previous study, there are nineteen cuproptosis regulators (NFE2L2, NLRP3, ATP7B, ATP7A, SLC31A1, FDX1, LIAS, LIPT1, LIPT2, DLD, DLAT, PDHA1, PDHB, MTF1, GLS, CDKN2A, DBT, GCSH, and DLST). After obtaining the expression matrix of 19 cuproptosis regulators, Pearson correlation analysis was performed (|Pearson R| > 0.4, *p* < 0.05) and we obtained 990 CRLs (Figure 2A). We randomly divided the patient data in TCGA into test cohort and training cohort, and the training group was imported to construct our model. To identify prognostic CRLs, we used the univariate Cox regression analysis (*p* < 0.05). The hazard ratio and expression of 10 prognostic CRLs were shown in Figure 2B.

**FIGURE 3:**
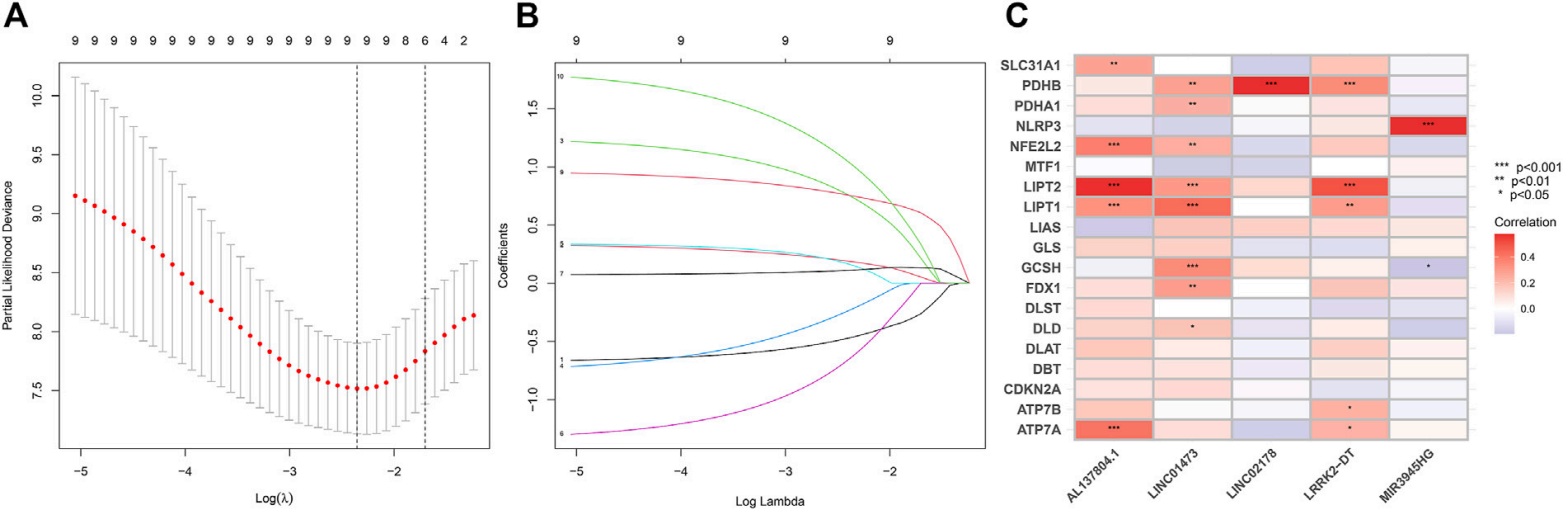
**(A–B)** LASSO Cox regression analysis between cuproptosis-related lncRNAs and HNSC patients. **(C)** Pearson correlation between 5 CRLs to build the prognostic model and 19 cuproptosis regulators. The asterisks indicated a statistically significant *p*-value (*p < 0.05; **p < 0.01; ***p < 0.001).

To construct the CRLs prognostic model in HNSC we performed the least absolute shrinkage and selection operator (LASSO) Cox regression (Figures 3A, B). As a result, 5 of 110 prognostic CRLs (*p* < 0.01) were filtered to build the prognostic model (Figure 3C). The following formula was used to compute each patient’s risk score: risk score = (0.506759183159314 * LINC02178 expression) + (1.7284811379188 * AL137804.1 expression) + (−1.98290017222121 * LINC01473 expression) + (1.00674378928319 * MIR3945HG expression) + (1.75380176645026 * LRRK2-DT expression).

### Validation of the CRLs prognostic model

**FIGURE 4:**
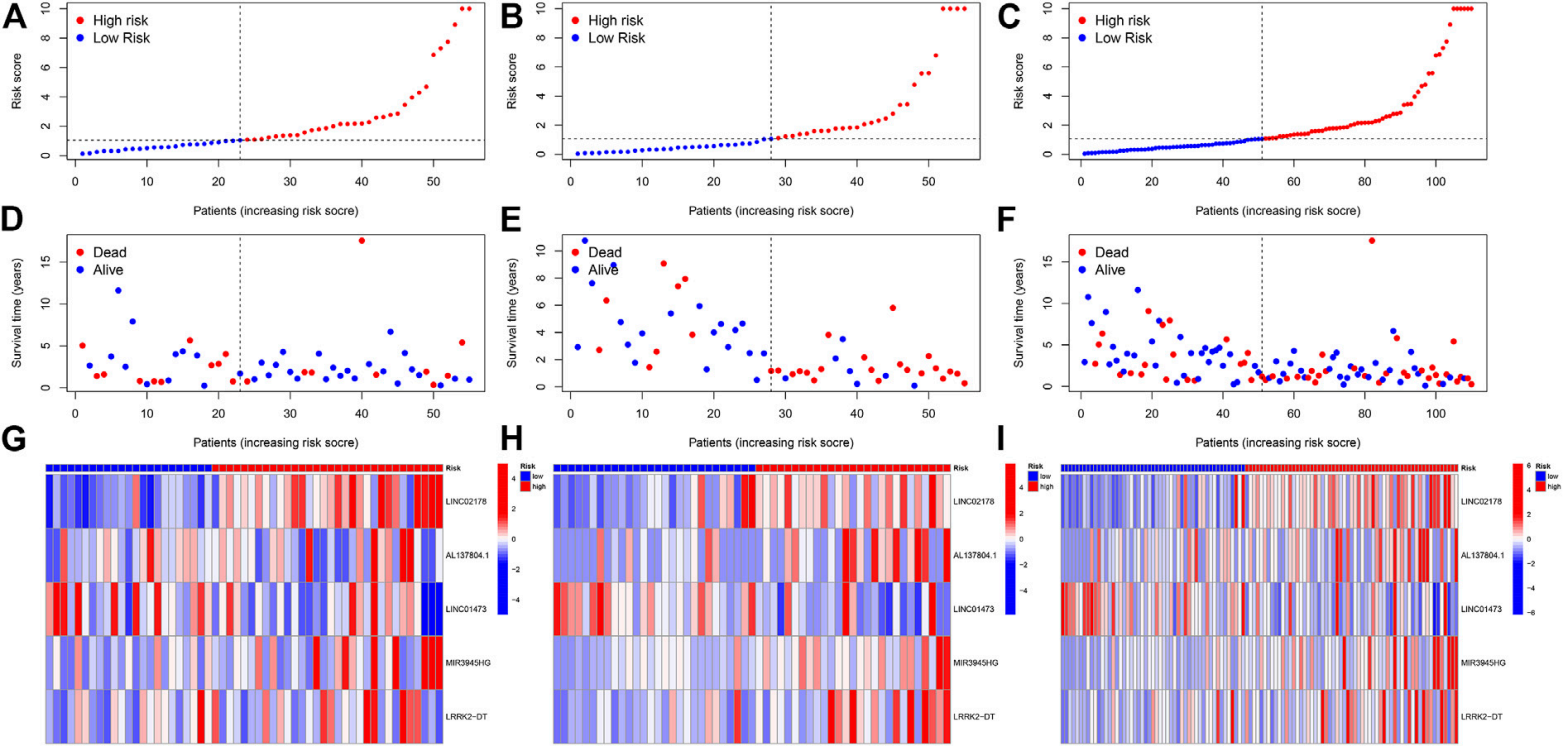
**(A–C)** Classification of patients as high or low risk based on median risk score. **(A)** is from the testing cohort; **(B)** is from the training cohort and **(C)** is from the entire set. **(D–F)** Distribution plot of the risk score and survival status of the patients with HNSC. **(D)** is from the testing cohort; **(E)** is from the training cohort and **(F)** is from the entire set. **(G–I)** Heatmap of correlation between patients in different risk groups and expression of 5 prognostic CRLs. **(G)** is from the testing cohort; **(H)** is from the training cohort and **(I)** is from the entire set.

In each cohort, according to the median value of the risk scores, the patients were divided into a low-risk group and a high-risk group (Figures 4A–I). As shown in the Figures 4A–F, the survival time of the patients with HNSC was longer in the low-risk group than in the high-risk group in both the training, testing sets, and entire set, respectively. Besides, the distribution plot of the risk score and survival status showed that the higher the risk score, the more deaths of the patients with HNSC. At the same time, we further studied the expression of lncRNA selected for our model construction in the high- and low-risk groups, which can be used for subsequent studies (Figures 4G–I).

**FIGURE 5:**
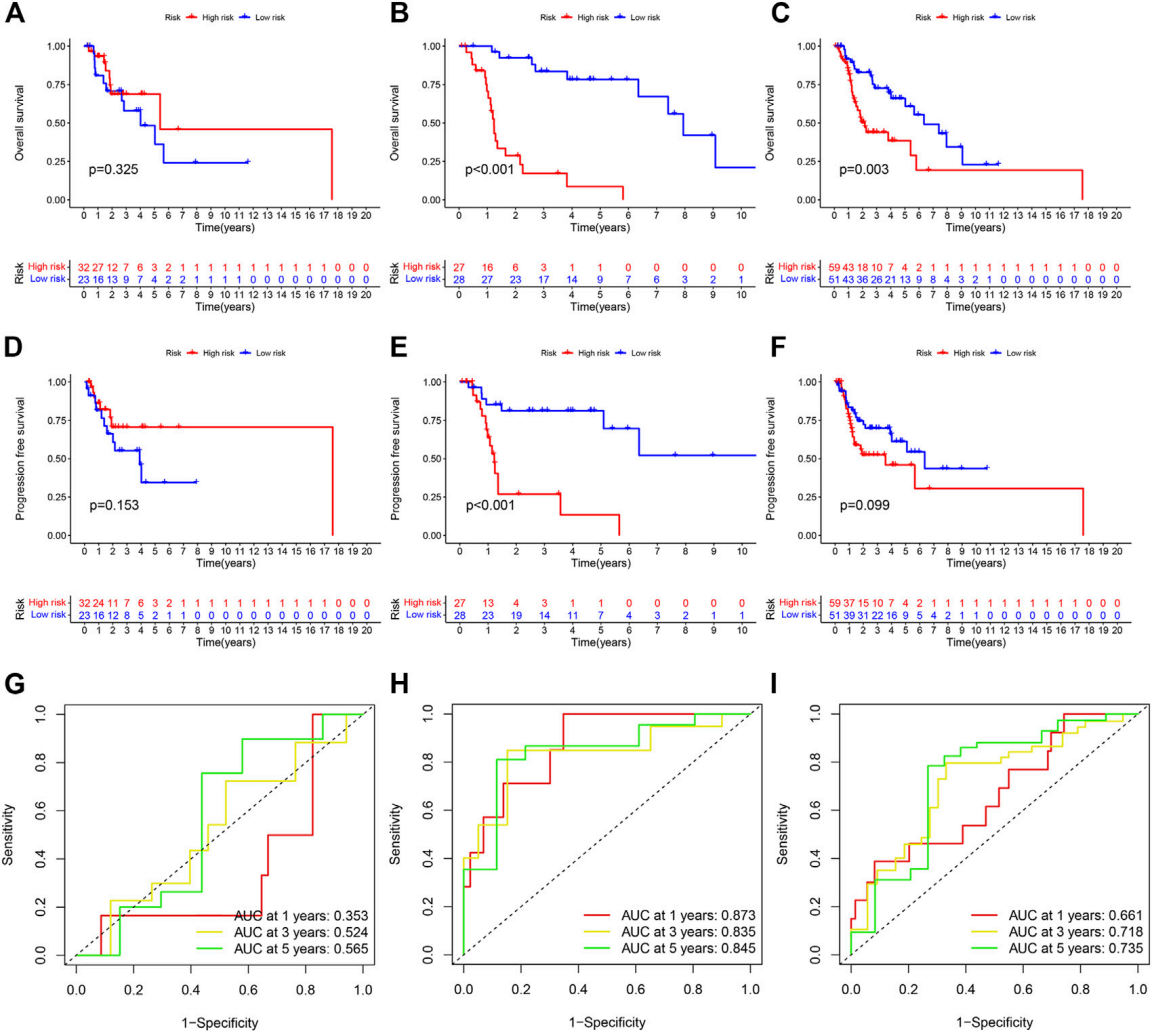
**(A–C)** Kaplan-Meier curve to show overall survival of patients in different risk groups. **(A)** is from the testing set; **(B)** is from the training set and **(C)** is from the entire set. **(D–F)** Kaplan-Meier curve to show progression-free survival of patients in in different risk groups. **(D)** is from the testing set; **(E)** is from the training set and **(F)** is from the entire set. **(G–I)** ROC curve to show the performance of CRLs risk model. **(G)** is from the testing set; **(H)** is from the training set and **(I)** is from the entire set.

According to the results, we found that in training set and entire set, the survival time of the patients with HNSC was significantly longer in the low-risk group than in the high-risk group (*p* < 0.05) (Figures 5A–C). Additionally, we found that progression-free survival (PFS) was also significantly shorter in the high-risk group than in the low-risk group (Figures 5D–F). In order to further test the predictive power of our model, we conducted time-dependent receiver operating characteristic (ROC) analysis in three cohort. The area under curve (AUC) of the ROC greater than 0.5 was considered to have good predictive capacity. In the test group, AUC values at years 3 and 5 were all greater than 0.5 (0.524 and 0.565, Figure 5G). In the training group, AUC values at years 1, 3, and 5 were all greater than 0.8 (0.873, 0.835, and 0.845, respectively, Figure 5H). In the entire cohort, AUC values at years 1, 3, and 5 were all greater than 0.6 (0.661, 0.718 and 0.735, respectively, Figure 5I).

### Independence analysis of the prognostic model

**FIGURE 6:**
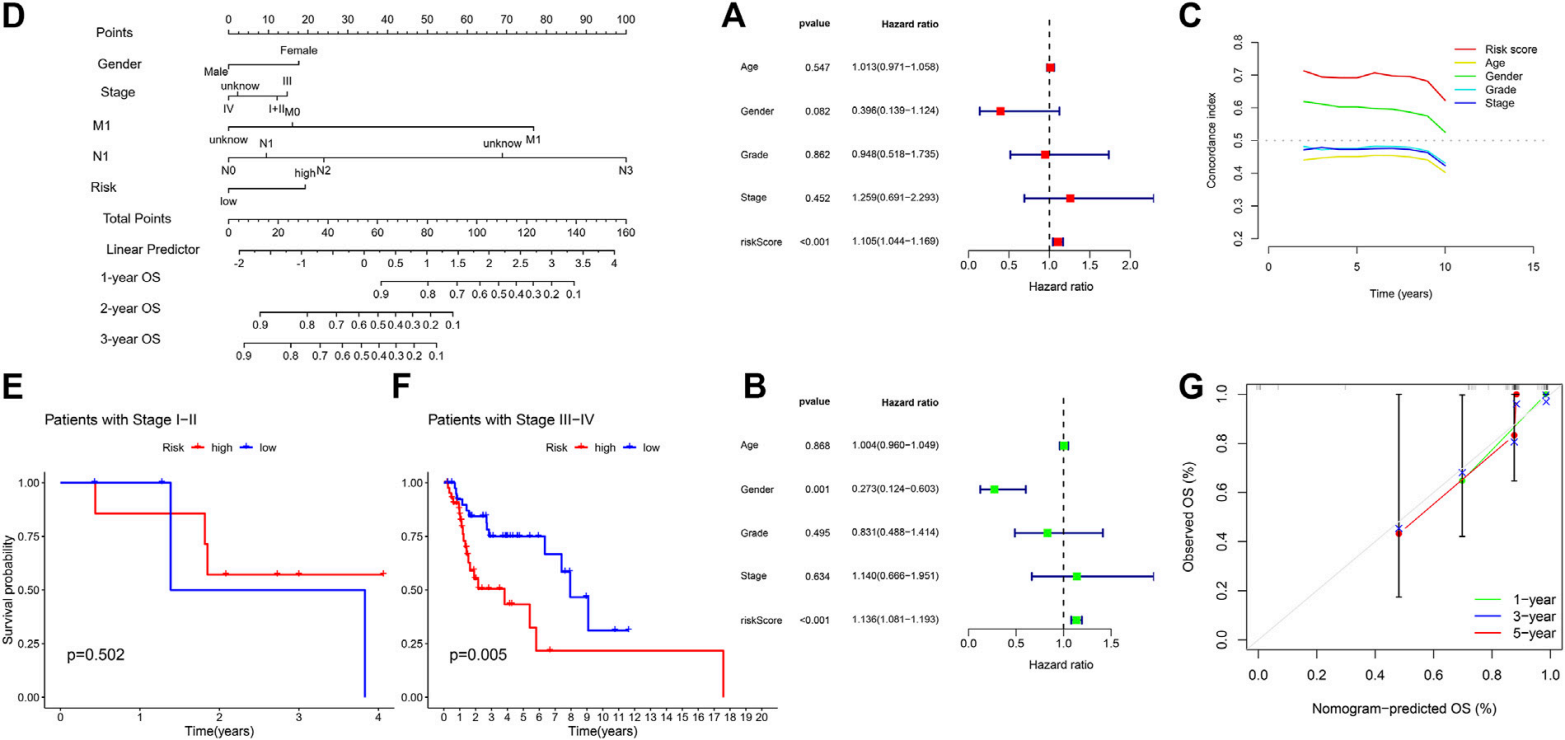
**(A)** Univariate Cox regression analysis between prognostic signatures and overall survival. **(B)** Multivariate cox regression analysis between prognostic signatures and overall survival. **(C)** The concordance index of different clinical characteristics and risk scores. **(D)** Nomogram of prognostic factors to predict the 1-, 3- and 5-years survival rates of patients with HNSC. **(E)** Kaplan-Meier curve to show overall survival of patients with early stage I-II tumor in different risk groups. **(F)** Kaplan-Meier curve to show overall survival of patients with late-stage III-IV tumor in different risk groups. **(G)** The calibration curves between the prediction by nomogram and actual survival.

Tofurther determine whether the signature of CRLs could be used as independent prognostic factors, independent of other clinical characteristics, we constructed univariate Cox regression and multivariate Cox regression analyses. As seen in Figure 6A, univariate Cox regression analysis showed that the prognostic signature of 5 CRLs was an independent variable to predict the outcome of OS in patients with HNSC (HR = 1.105, 95% CI, 1.044–1.169, *p* < 0.001). Multiple Cox regression analysis also revealed that the prognostic signature of 5 CRLs was an independent prognosis factor for HNSC (HR = 1.136, 95% CI, 1.081–1.193, *p* < 0.001) after adjusting for gender, age, grade and stage (Figure 6B). Furthermore, the concordance index (C-index) of the risk score was higher than that of clinical characteristics, including age, gender, grade, and stage (Figure 6C).

Subsequently, we established a nomogram using these independent prognostic factors (TNM stage, grade and risk score) to predict the 1-, 3- and 5-years survival rates of patients with HNSC (Figure 6D). Particularly in stage of tumor, we found our model performed better in patients with late-stage tumor, i.e., stage III-IV, while there was no significance in terms of patients with early-stage tumor (Figures 6E, F). Such difference showed in K-M curve could account for the 1-year AUC in our test group since the CRL model was inclined to predict late-stage HNSC. In addition, we further developed calibration curves to verify the effectiveness of nomogram model for predicting the survival rates for patients with HNSC at 1, 3, and 5 years. The results showed that the calibration curves presented an optimal agreement between the prediction by nomogram and actual survival (Figure 6G). In brief, it was of great significance that the nomogram had the potential to predict the survival outcomes for patients with HNSC.

### Principal component analysis (PCA) and functional enrichment analysis

**FIGURE 7:**
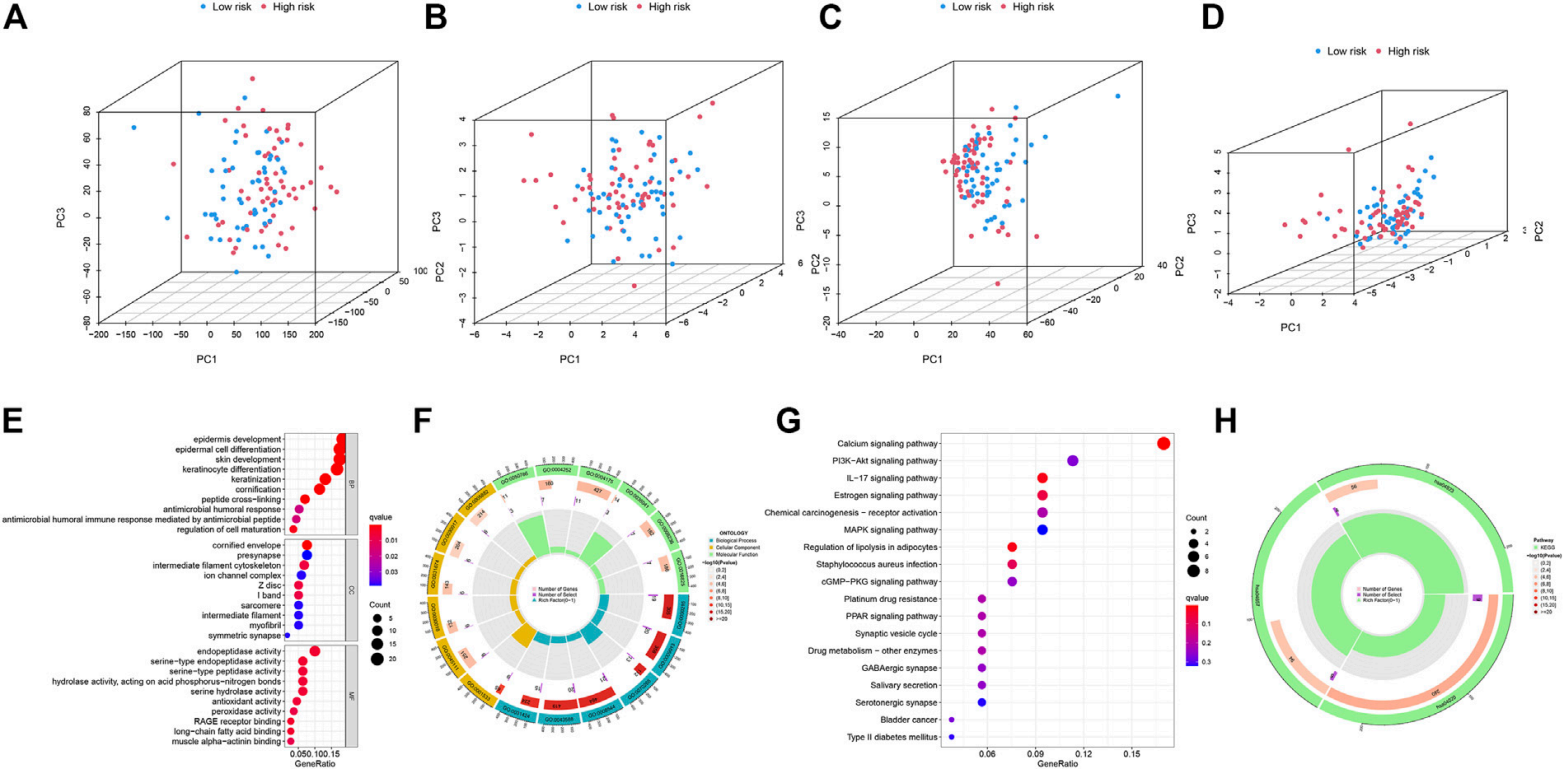
**(A–D)** PCA analysis of patients in low- and high-risk groups based on four expression profiles. **(A)** is from the entire gene expression profiles; **(B)** is from cuproptosis genes; **(C)** is from cuproptosis-related lncRNAs and **(D)** is from the 5 CRLs that established the risk model. **(E–F)** GO enrichment analysis of DEGs between the low- and high-risk groups in TCGA-HNSC. **(G–H)** KEGG enrichment analysis of DEGs between the low- and high-risk groups in TCGA-HNSC.

To examine the differences and distinction between the low- and high-risk groups, we implemented PCA based on the four expression profiles (entire gene expression profiles, cuproptosis genes, CRLs and the risk signature established by the expression profiles of the 5 CRLs). The results showed that 5 CRLs possessed a good discrimination ability to distinguish between the low- and high-risk groups (Figures 7A–D).

Then, we identified 177 DEGs between the low- and high-risk groups of the TCGA set (|log2FoldChange| > 1, *p* < 0.05). We further performed functional enrichment analyses to elucidate the biological functions of DEGs between the two groups. The GO analyses demonstrated significant enrichment of epidermal cell differentiation (Figures 7E, F). The KEGG analysis revealed enrichment in several pathways associated with “Calcium signaling pathway,” “IL−17 signaling pathway” and “Regulation of lipolysis in adipocytes” (Figures 7G, H). These results suggested that CRLs signature are involved in the development and progression of HNSC.

### Examination of immune signatures related to CRLs

**FIGURE 8:**
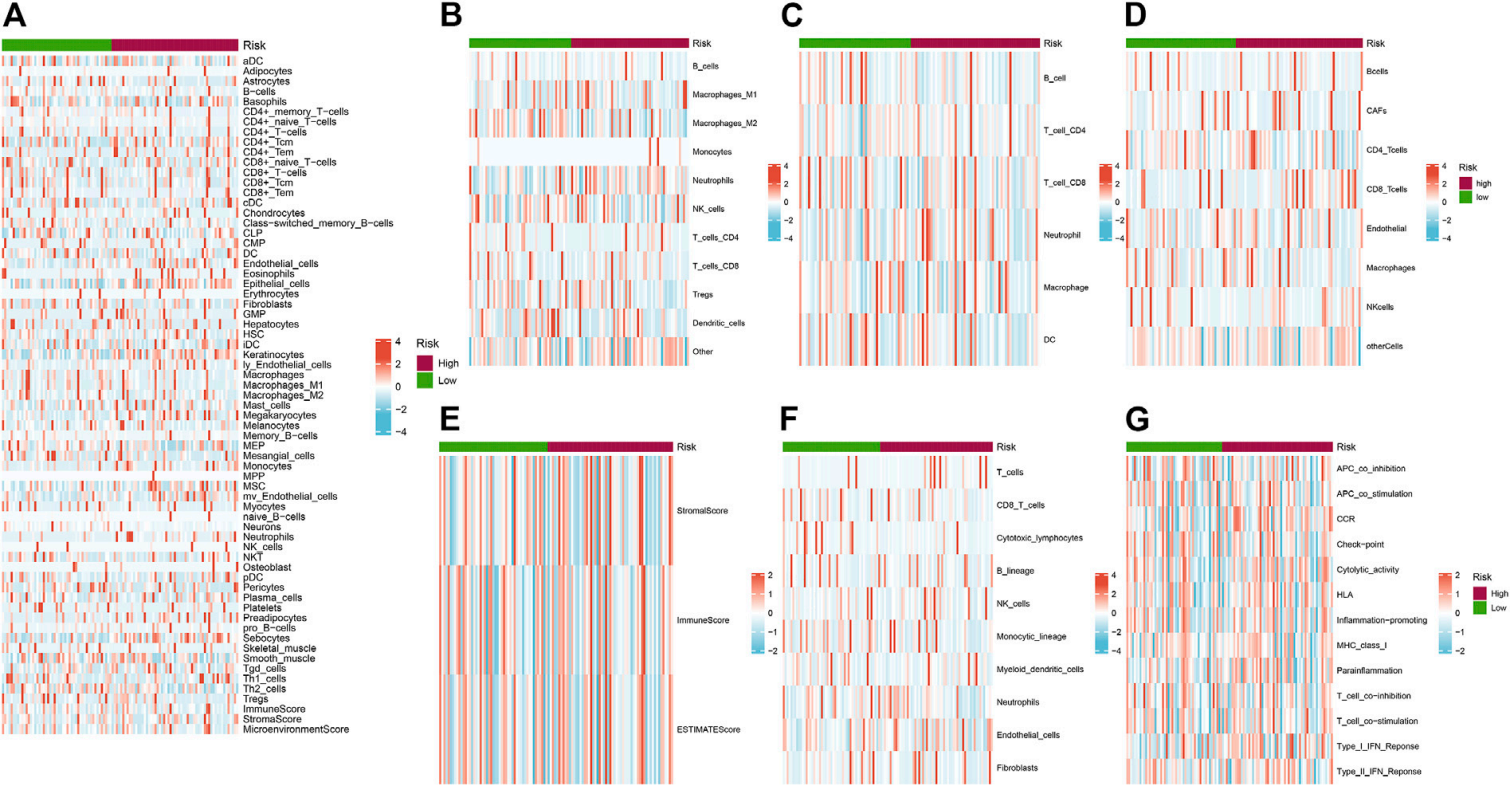
**(A–G)** Heatmaps of the immune infiltration related to different risk groups. **(A)** XCELL; **(B)** QUANTISEQ; **(C)** TIMER; **(D)** EPIC; **(E)** ESTIMATE; **(F)** MCPCOUNTER and **(G)** ssGSEA. Red and blue indicate the infiltration value calculated by the corresponding algorithm.

We depicted the heatmaps of the immune infiltration related to gene expression of high and low risk groups in 7 different algorithms including XCELL, QUANTISEQ, TIMER, EPIC, ESTIMATE, MCPCOUNTER and ssGSEA (Figures 8A–G). We found high- and low-risk groups of HNSC showed distinct infiltration signatures in terms of different immune or stromal cells.

### TMB and TIDE analysis

**FIGURE 9:**
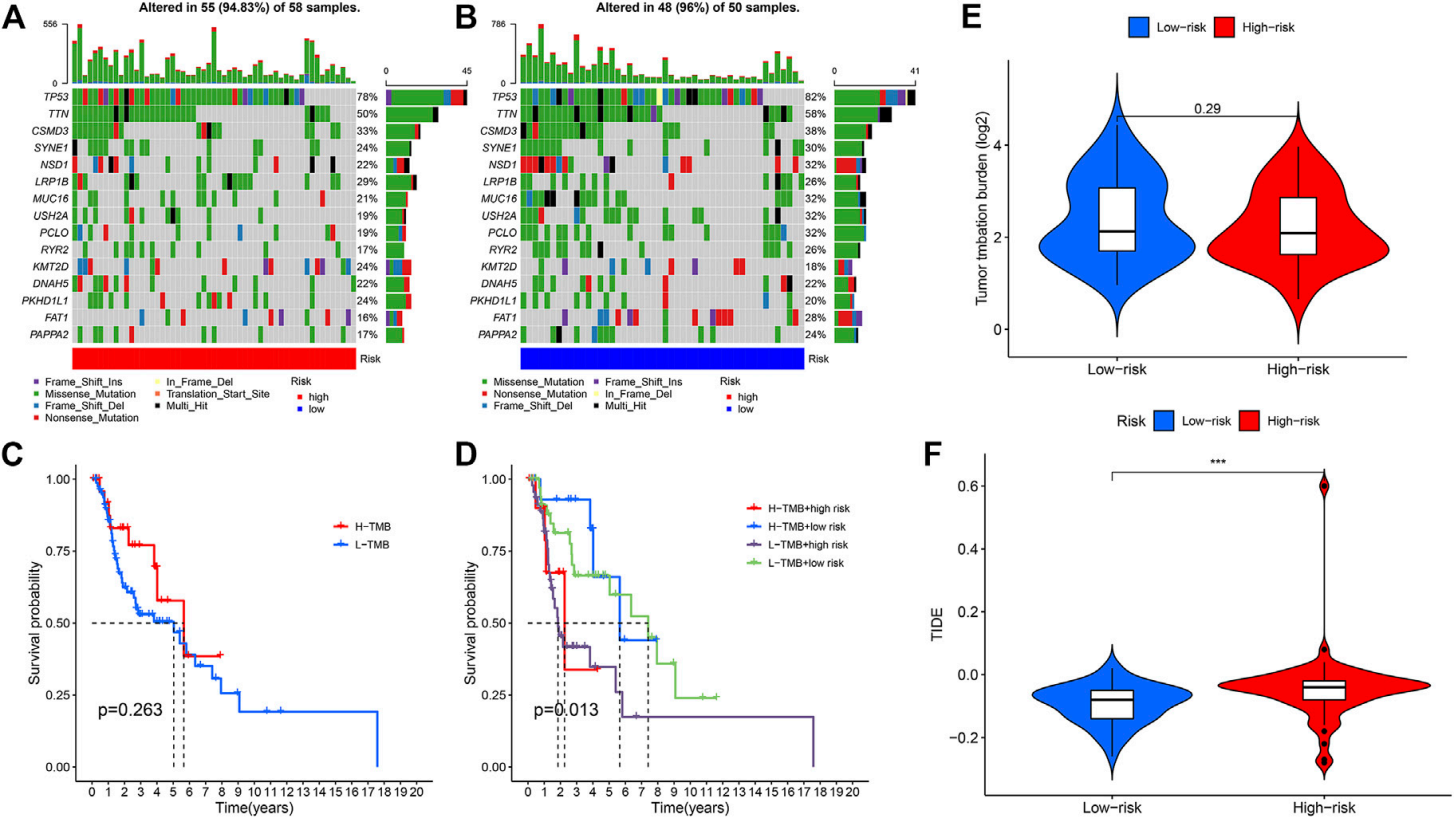
**(A)** Mutation rates and types of top 15 genes in the high-risk group. **(B)** Mutation rates and types of top 15 genes in the low-risk group. **(C)** Kaplan-Meier curve to show survival of patients in different TMB groups. **(D)** Kaplan-Meier curve to show survival of patients in different TMB and risk groups. **(E)** TMB between high and low risk groups. **(F)** TIDE value between high and low risk groups (***p < 0.001).

Increasing evidence suggest that tumour mutation burden status is associated with clinical response to immunotherapy in the HNSC. So, we identified the TMB-specific genes between high- and low-risk groups by the R package “maftools”. The results showed that the frequency of mutations in the low-risk group and the high-risk group among the top 15 genes with the highest mutation rates (Figures 9A, B). Subsequently, we divided the patients into high TMB group and low TMB group according to the TMB score. The survival analysis showed that the high TMB group had a higher survival rate than the low TMB group without significance in statistics (Figure 9C). We further evaluated the synergistic effect of TMB and CRLs-scores groups in prognostic stratification (Figure 9D). Stratified survival analysis showed that patients with low-risk scores possessed a significantly better prognosis than patients with high-risk scores despite of TMB level. Our analysis of TMB between different risk groups showed no significance (Figure 9E), which was consistent with aforementioned results. Nevertheless, the TIDE scores were significantly higher in the high-risk group compared to the low-risk group (Figure 9F).

### External validation of CRL prediction model

**FIGURE 10:**
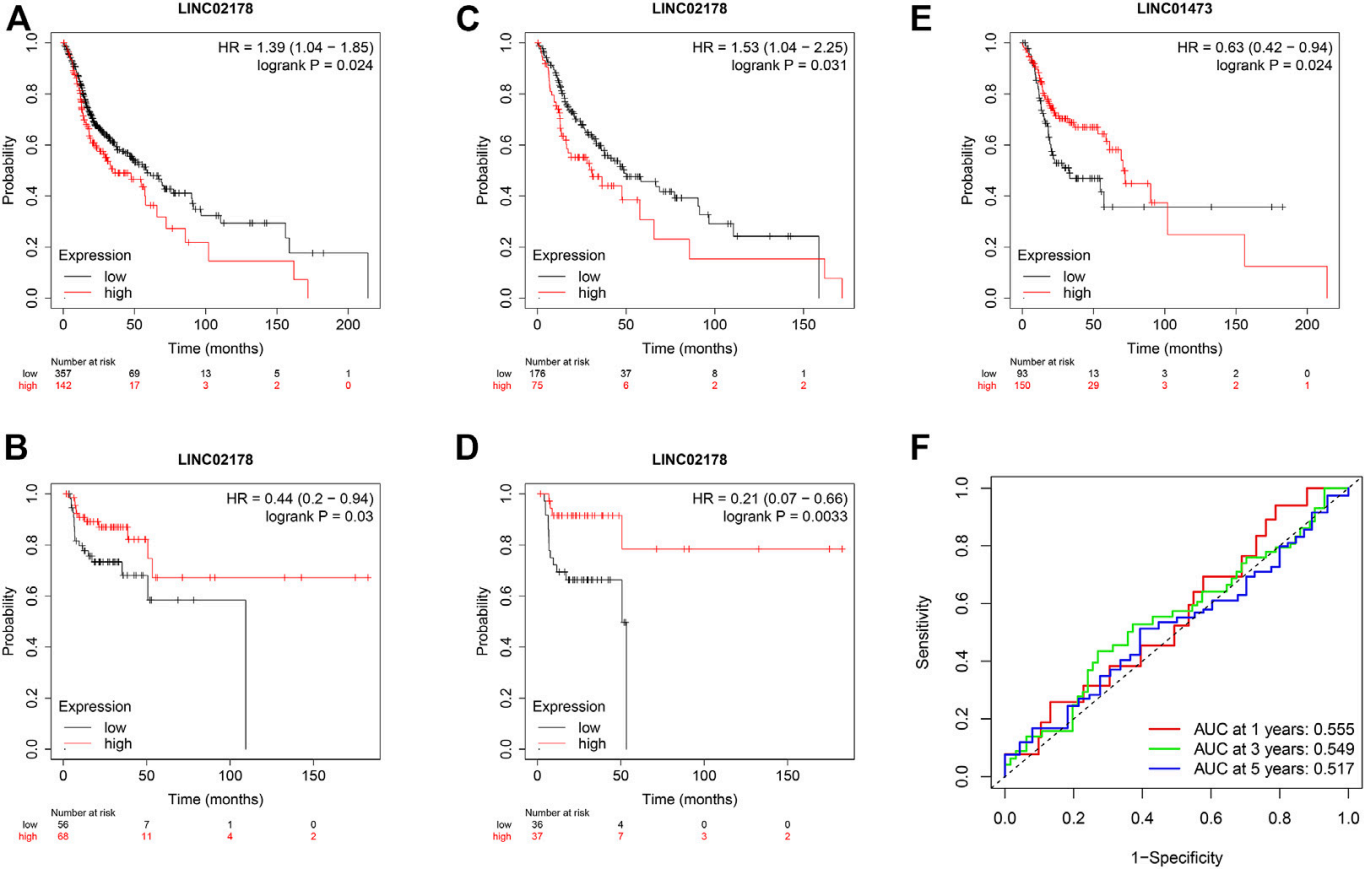
**(A–E)** Prognostic value of LINC02178 and LINC01473 based on Kaplan–Meier plotter. **(A)** Kaplan-Meier curve to show OS of patients in different LINC02178 expression levels. **(B)** Kaplan-Meier curve to show RFS of patients in different LINC02178 expression levels. **(C)** Kaplan-Meier curve to show OS of patients with high-TMB in different LINC02178 expression levels. **(D)** Kaplan-Meier curve to show RFS of patients with low-TMB in different LINC02178 expression levels. **(E)** Kaplan-Meier curve to show OS of patients with low-TMB in different LINC01473 expression levels. **(F)** Prognostic value of CRLs based on TCGA-CESC.

Then, we examined the prognostic value of LINC02178 and LINC01473 in the external Kaplan–Meier Plotter database (Figures 10A–E). We found that LINC02718 was significantly correlated with OS and recurrence free survival (RFS). When we divided samples into groups of high-TMB and low-TMB, LINC02718 showed significant association with OS in high-TMB group and that with RFS in low-TMB group. LINC01473 was dramatically correlated with OS in low-TMB group, which reconfirm the prognostic role of CRLs was independent of TMB level. The results of the survival analysis were consistent with previous outcomes.

Moreover, considering that cervical squamous cell carcinoma and endocervical adenocarcinoma (CESC) also belongs to the highly HPV-related type of cancer, we conducted ROC analysis as well based on external TCGA-CESC database to evaluated the generalization of the CRL model (Figure 10F). The result showed that our model also possessed effective prediction in CESC, indicating the reliability of CRLs as prognostic biomarkers.

### Experimental validation of CRL model as potential biomarker

**FIGURE 11:**
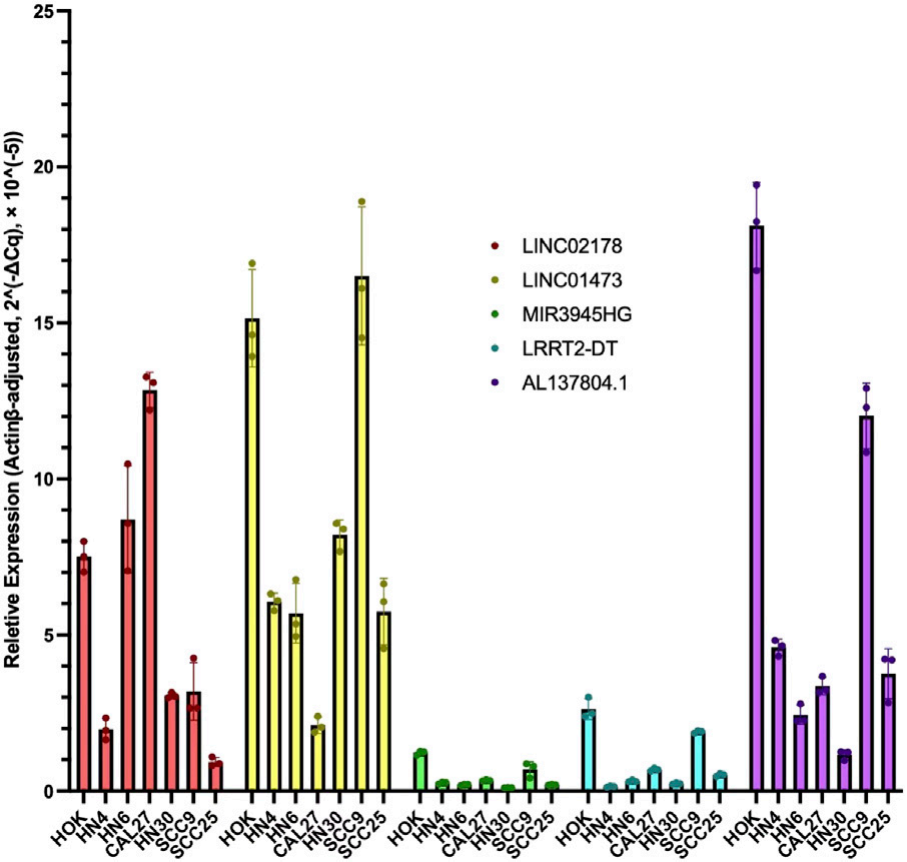
RT-qPCR results of LINC02178, AL137804.1, LINC01473, MIR3945HG, and LRRK2-DT in different kinds of HNSC cells compared to human oral keratinocytes.

To further validate the prognostic value of our CRL model, we performed qRT-PCR experiments to illustrate the expression signature of the 5 CRLs which were found in LASSO Cox analysis to construct the risk model. The results in qRT-PCR indicated an overall signature of differential expression levels of LINC02178, AL137804.1, LINC01473, MIR3945HG, and LRRK2-DT. In different kinds of HNSC cells, they were differentially expressed compared to human oral keratinocytes (Figure 11), which matched the results of our previous bioinformatics analysis based on public databases.

## Discussion

Head and neck cancer is the sixth most frequent malignant tumor worldwide and more than 90% of these cancers are HNSC (Liu et al., 2018). Over the last several decades, smoking-related HNSC has decreased whereas HPV-related HNSC has become more and more common (McDermott and Bowles, 2019). The immune system plays an important role in head and neck carcinogenesis (Varilla et al., 2013). Certain subsets of HNSC are immunosuppressive human malignancies, marked by T-cell dysfunction, low levels of CD4^+^ and CD8^+^ T-cell, increased T-regulatory cells, cytokine alterations and antigen presentation defects (Sun et al., 2014). Such immunosuppressive environment was responsible for the unsatisfactory effects of immune checkpoint inhibitors such as PD-1 immunotherapy (Mandal et al., 2016), so the improved therapeutic approaches are needed. By researching the regulatory role of cuproptosis in HNSC, the novel molecules signature of the diagnosis and treatment could bring new strategies for physicians.

The results of our study revealed that there were 10 CRLs influenced the survival of the patients with HNSC, and 5 of them (LINC02178, AL137804.1, LINC01473, MIR3945HG, LRRK2-DT) were selected to establish the prognostic signature. Studies found LINC02178 was related to autophagy in BLCA and necroptosis in UCEC (Sun et al., 2020; Lin et al., 2022), and it was a prognostic predictor in LUAD (Li et al., 2018). Yang et al. investigated a novel risk model with AL137804.1 for predicting the prognosis of bladder cancer (Yang et al., 2019), and Zhang et al. also described AL137804.1 as a crucial lncRNA in bladder urothelial carcinoma (Zhang et al., 2020a). Aberrant expression of LINC01473 in osteoblasts facilitated imbalanced bone formation and resorption in multiple myeloma (MM), which influenced immune escape of MM (Peng et al., 2022). It has been reported that MIR3945HG was upregulated in macrophages infected with *Mycobacterium tuberculosis* or HIV (Yang et al., 2016; Schynkel et al., 2020). In addition, the expression of MIR3945HG has been confirmed to be downregulated in LUAD and LUSC based on both bioinformatics and qRT-PCR validation (Chen et al., 2017; Wang et al., 2019). However, the function of LRRK2-DT has not been reported yet.

In both training sets and testing sets, CRL model showed the robust capacity in predicting survival outcomes of patients with HNSC. The results of ROC curve, Kaplan–Meier survival analysis, univariate and multivariate Cox regression analysis revealed that the risk score of CRLs signature could be used as an independent factor to predict poor prognosis in HNSC. The C-index of our risk score was higher than other metrics, which reflected better performance of our model. In addition, our nomogram and 1-, 3-, and 5-year calibration curves demonstrated an optimal consistency between the prediction by nomogram and actual survival. PCA analysis showed the ability of 5 CRLs to distinguish between high- and low-risk groups. Moreover, we utilized the DEGs between two groups to explore the biological functions and pathways through enrichment analysis in GO and KEGG database. Metabolic and immune pathways were involved in the difference risk score based on CRLs, which was similar to the results of other cell death processes (Kimmelman and White, 2017; Pavlyukov et al., 2018; Bebber et al., 2020; Zhang et al., 2021). Our result indicated that cuproptosis could be associated with calcium, IL-17, adipocytes and related pathways. Moreover, there may be crosstalk between cuproptosis and other processes like autophagy or ferroptosis. We observed that the proportion of gene mutations was similar between two groups, which could be responsible for the non-significant differences between high-TMB group and low-TMB group since TMB is described as the number of somatic mutations in tumor biopsy (Aggarwal et al., 2020).

TMB has been shown to correlate with response to immunotherapy as a biomarker. Given the growing economic burden and toxic side effects of common cancer treatments, more robust and economic biomarkers which can predict the response to immunotherapy should be investigated. It has been proved TMB level partially predicts the response to treatment, but its predictive value may be weakened by many factors (Thorsson et al., 2018). In HNSC, it was reported that TMB was related to B and CD4^+^ T-cell infiltration, and affected the prognosis (Zhang et al., 2020b). However, in the most informative study which evaluated TMB in pan-cancer, researchers did not find any prognostic impact of TMB in HNSC (Wu et al., 2019). Which factors influenced the prognostic performance of TMB in HNSC requires further study. In our study, like Figures 9C–9E, we found TMB was not a robust predictor of prognosis, while our risk model showed a significant difference between high- and low groups in addition to TMB level.

Previous studies have found CRLs could perform as prognostic biomarkers in different kinds of tumors (Huili et al., 2022; Xu et al., 2022; Yang et al., 2022), indicating the important role of CRLs in cancer. Nevertheless, the detailed comparison between CRLs and other indices was insufficient. In our study, we found CRLs possessed complex immune signature, and predicted the prognosis independent of TMB. We also performed two-way verifications based on external datasets and qRT-PCR to increase the validation of our model. In both immune infiltration analysis and qPCR verification, high- and low-risk groups showed various correlation with CRLs, suggesting the complicated underlying mechanisms for prognostic CRLs. Compared to human oral keratinocytes, the 5 CRLs were significantly differently expressed in qPCR. Nevertheless, the expression signature in different HNSC cell lines were not the same, indicating the heterogeneity of different kinds of HNSC as well as the versatility of CRLs. In addition, our results provided new insights that CRL model can not only work in HNSC, but also be generalized in related tumors such as CESC.

In conclusion, our study investigated the prognostic value of these CRLs in HNSC, and all of the results proved our risk signature was highly robust and effective for predicting the prognosis of the patients with HNSC. Expression of CRLs were closely correlated with tumor immunity. To explore the detailed regulatory effects of CRLs in HNSC, further investigation and experiments are needed.

## Data Availability

The original contributions presented in the study are included in the article/supplementary material, further inquiries can be directed to the corresponding authors.

## References

[B1] Aggarwal C. Cohen R. B. Morrow M. P. Kraynyak K. A. Sylvester A. J. Knoblock D. M. (2019). Immunotherapy targeting HPV16/18 generates potent immune responses in HPV-associated head and neck cancer. Clin. Cancer Res. 25 (1), 110–124. 10.1158/1078-0432.CCR-18-1763 30242022 PMC6320307

[B2] Aggarwal C. Thompson J. C. Chien A. L. Quinn K. J. Hwang W-T. Black T. A. (2020). Baseline plasma tumor mutation burden predicts response to pembrolizumab-based therapy in patients with metastatic non-small cell lung cancer. Clin. Cancer Res. 26 (10), 2354–2361. 10.1158/1078-0432.CCR-19-3663 32102950 PMC7231655

[B3] Bauml J. Seiwert T. Y. Pfister D. G. Worden F. Liu S. V. Gilbert J. (2017). Pembrolizumab for platinum-and cetuximab-refractory head and neck cancer: Results from a single-arm, phase II study. J. Clin. Oncol. 35 (14), 1542–1549. 10.1200/JCO.2016.70.1524 28328302 PMC5946724

[B4] Bebber C. M. Müller F. Prieto Clemente L. Weber J. von Karstedt S. (2020). Ferroptosis in cancer cell biology. Cancers 12 (1), 164. 10.3390/cancers12010164 31936571 PMC7016816

[B5] Chen W-J. Tang R-X. He R-Q. Li D-Y. Liang L. Zeng J-H. (2017). Clinical roles of the aberrantly expressed lncRNAs in lung squamous cell carcinoma: A study based on RNA-sequencing and microarray data mining. Oncotarget 8 (37), 61282–61304. 10.18632/oncotarget.18058 28977863 PMC5617423

[B6] Degenhardt K. Mathew R. Beaudoin B. Bray K. Anderson D. Chen G. (2006). Autophagy promotes tumor cell survival and restricts necrosis, inflammation, and tumorigenesis. Cancer Cell 10 (1), 51–64. 10.1016/j.ccr.2006.06.001 16843265 PMC2857533

[B7] Ferris R. L. Blumenschein G. Jr Fayette J. Guigay J. Colevas A. D. Licitra L. (2016). Nivolumab for recurrent squamous-cell carcinoma of the head and neck. N. Engl. J. Med. 375, 1856–1867. 10.1056/NEJMoa1602252 27718784 PMC5564292

[B8] Huili Y. Nie S. Zhang L. Yao A. Liu J. Wang Y. (2022). Cuproptosis-related lncRNA: Prediction of prognosis and subtype determination in clear cell renal cell carcinoma. Front. Genet. 13, 958547. 10.3389/fgene.2022.958547 36072656 PMC9441767

[B9] Jiang P. Gu S. Pan D. Fu J. Sahu A. Hu X. (2018). Signatures of T cell dysfunction and exclusion predict cancer immunotherapy response. Nat. Med. 24 (10), 1550–1558. 10.1038/s41591-018-0136-1 30127393 PMC6487502

[B10] Kimmelman A. C. White E. (2017). Autophagy and tumor metabolism. Cell metab. 25 (5), 1037–1043. 10.1016/j.cmet.2017.04.004 28467923 PMC5604466

[B11] Li T. Fan J. Wang B. Traugh N. Chen Q. Liu J. S. (2017). Timer: A web server for comprehensive analysis of tumor-infiltrating immune cells. Cancer Res. 77 (21), e108–e110. 10.1158/0008-5472.CAN-17-0307 29092952 PMC6042652

[B12] Li Y. Y. Yang C. Zhou P. Zhang S. Yao Y. Li D. (2018). Genome‐scale analysis to identify prognostic markers and predict the survival of lung adenocarcinoma. J. Cell. Biochem. 119 (11), 8909–8921. 10.1002/jcb.27144 30105759

[B13] Lin Z. Fan W. Sui X. Wang J. Zhao J. (2022). Necroptosis-related LncRNA signatures for prognostic prediction in uterine corpora endometrial cancer. Reprod. Sci., 1–14. 10.1007/s43032-022-01023-9 PMC9988759 35854199

[B14] Liu C. Guo T. Xu G. Sakai A. Ren S. Fukusumi T. (2018). Characterization of alternative splicing events in HPV-negative head and neck squamous cell carcinoma identifies an oncogenic DOCK5 variant. Clin. Cancer Res. 24 (20), 5123–5132. 10.1158/1078-0432.CCR-18-0752 29945995 PMC6440699

[B15] Ludwig S. Floros T. Theodoraki M-N. Hong C-S. Jackson E. K. Lang S. (2017). Suppression of lymphocyte functions by plasma exosomes correlates with disease activity in patients with head and neck cancer. Clin. Cancer Res. 23 (16), 4843–4854. 10.1158/1078-0432.CCR-16-2819 28400428 PMC5559308

[B16] Luo X. Qiu Y. Jiang Y. Chen F. Jiang L. Zhou Y. (2018). Long non-coding RNA implicated in the invasion and metastasis of head and neck cancer: Possible function and mechanisms. Mol. cancer 17 (1), 14–16. 10.1186/s12943-018-0763-7 29368602 PMC5784721

[B17] Mandal R. Şenbabaoğlu Y. Desrichard A. Havel J. J. Dalin M. G. Riaz N. (2016). The head and neck cancer immune landscape and its immunotherapeutic implications. JCI insight 1 (17), e89829. 10.1172/jci.insight.89829 27777979 PMC5070962

[B18] McDermott J. D. Bowles D. W. (2019). Epidemiology of head and neck squamous cell carcinomas: Impact on staging and prevention strategies. Curr. Treat. options Oncol. 20 (5), 43–13. 10.1007/s11864-019-0650-5 31011837

[B19] Nagy Á. Munkácsy G. Győrffy B. (2021). Pancancer survival analysis of cancer hallmark genes. Sci. Rep. 11 (1), 6047. 10.1038/s41598-021-84787-5 33723286 PMC7961001

[B20] Pavlyukov M. S. Yu H. Bastola S. Minata M. Shender V. O. Lee Y. (2018). Apoptotic cell-derived extracellular vesicles promote malignancy of glioblastoma via intercellular transfer of splicing factors. Cancer Cell 34 (1), 119–135. 10.1016/j.ccell.2018.05.012 29937354 PMC6048596

[B21] Peng F. Yan S. Liu H. Liu Z. Jiang F. Cao P. (2022). Roles of LINC01473 and CD74 in osteoblasts in multiple myeloma bone disease. J. Investigative Med. 70, 1301–1307. 10.1136/jim-2021-002192 PMC9240337 35145037

[B22] Peng W-X. Koirala P. Mo Y-Y. (2017). LncRNA-mediated regulation of cell signaling in cancer. Oncogene 36 (41), 5661–5667. 10.1038/onc.2017.184 28604750 PMC6450570

[B23] Schynkel T. Szaniawski M. A. Spivak A. M. Bosque A. Planelles V. Vandekerckhove L. (2020). Interferon-mediated long non-coding RNA response in macrophages in the context of HIV. Int. J. Mol. Sci. 21 (20), 7741. 10.3390/ijms21207741 33086748 PMC7589721

[B24] Sun W. Li W-J. Wu C-Y. Zhong H. Wen W-P. (2014). CD45RA-Foxp3high but not CD45RA+ Foxp3low suppressive T regulatory cells increased in the peripheral circulation of patients with head and neck squamous cell carcinoma and correlated with tumor progression. J. Exp. Clin. Cancer Res. 33 (1), 35–10. 10.1186/1756-9966-33-35 24761979 PMC4022051

[B25] Sun Z. Jing C. Xiao C. Li T. (2020). An autophagy-related long non-coding RNA prognostic signature accurately predicts survival outcomes in bladder urothelial carcinoma patients. Aging (Albany NY) 12 (15), 15624–15637. 10.18632/aging.103718 32805727 PMC7467376

[B26] Thorsson V. Gibbs D. L. Brown S. D. Wolf D. Bortone D. S. Yang T-H. O. (2018). The immune landscape of cancer. Immunity 48 (4), 812–830. e14. 10.1016/j.immuni.2018.03.023 29628290 PMC5982584

[B27] Tsvetkov P. Coy S. Petrova B. Dreishpoon M. Verma A. Abdusamad M. (2022). Copper induces cell death by targeting lipoylated TCA cycle proteins. Science 375 (6586), 1254–1261. 10.1126/science.abf0529 35298263 PMC9273333

[B28] Varilla V. Atienza J. Dasanu C. A. (2013). Immune alterations and immunotherapy prospects in head and neck cancer. Expert Opin. Biol. Ther. 13 (9), 1241–1256. 10.1517/14712598.2013.810716 23789839

[B29] Wang Y. Fu J. Wang Z. Lv Z. Fan Z. Lei T. (2019). Screening key lncRNAs for human lung adenocarcinoma based on machine learning and weighted gene co-expression network analysis. Cancer Biomarkers 25 (4), 313–324. 10.3233/CBM-190225 31322548 PMC12828841

[B30] Wu H-X. Wang Z-X. Zhao Q. Chen D-L. He M-M. Yang L-P. (2019). Tumor mutational and indel burden: A systematic pan-cancer evaluation as prognostic biomarkers. Ann. Transl. Med. 7 (22), 640. 10.21037/atm.2019.10.116 31930041 PMC6944566

[B31] Xu S. Liu D. Chang T. Wen X. Ma S. Sun G. (2022). Cuproptosis-associated lncRNA Establishes new prognostic profile and predicts immunotherapy response in clear cell renal cell carcinoma. Front. Genet. 13, 938259. 10.3389/fgene.2022.938259 35910212 PMC9334800

[B32] Yang F. Hong K. Zhao G. Liu C. Song Y. Ma L. (2019). Construction of prognostic model and identification of prognostic biomarkers based on the expression of long non-coding RNA in bladder cancer via bioinformatics. Beijing da xue xue bao Yi xue ban= J. Peking Univ. Health Sci. 51 (4), 615–622. 10.19723/j.issn.1671-167X.2019.04.003 PMC7433490 31420610

[B33] Yang G. Lu X. Yuan L. (2014). LncRNA: A link between RNA and cancer. Biochimica Biophysica Acta (BBA)-Gene Regul. Mech. 1839 (11), 1097–1109. 10.1016/j.bbagrm.2014.08.012 25159663

[B34] Yang L. Yu J. Tao L. Huang H. Gao Y. Yao J. (2022). Cuproptosis-related lncRNAs are biomarkers of prognosis and immune microenvironment in head and neck squamous cell carcinoma. Front. Genet. 13, 947551. 10.3389/fgene.2022.947551 35938003 PMC9354258

[B35] Yang X. Yang J. Wang J. Wen Q. Wang H. He J. (2016). Microarray analysis of long noncoding RNA and mRNA expression profiles in human macrophages infected with *Mycobacterium tuberculosis* . Sci. Rep. 6, 38963. 10.1038/srep38963 27966580 PMC5155227

[B36] Zhang C. Li Z. Hu J. Qi F. Li X. Luo J. (2020). Identification of five long noncoding RNAs signature and risk score for prognosis of bladder urothelial carcinoma. J. Cell. Biochem. 121 (1), 856–866. 10.1002/jcb.29330 31373406

[B37] Zhang L. Li B. Peng Y. Wu F. Li Q. Lin Z. (2020). The prognostic value of TMB and the relationship between TMB and immune infiltration in head and neck squamous cell carcinoma: A gene expression-based study. Oral Oncol. 110, 104943. 10.1016/j.oraloncology.2020.104943 32919362

[B38] Zhang M-Y. Huo C. Liu J-Y. Shi Z-E. Zhang W-D. Qu J-J. (2021). Identification of a five autophagy subtype-related gene expression pattern for improving the prognosis of lung adenocarcinoma. Front. Cell Dev. Biol. 9, 756911. 10.3389/fcell.2021.756911 34869345 PMC8636677

